# Basolateral amygdala nucleus responses to appetitive conditioned stimuli correlate with variations in conditioned behaviour

**DOI:** 10.1038/ncomms12275

**Published:** 2016-07-22

**Authors:** Seung-Chan Lee, Alon Amir, Drew B. Headley, Darrell Haufler, Denis Pare

**Affiliations:** 1 Center for Molecular and Behavioral Neuroscience, Rutgers State University, Newark, New Jersey 07102, USA

## Abstract

In the lateral amygdala (LA), training-induced increases in neuronal responsiveness to conditioned stimuli (CSs) reflect potentiated sensory responses that drive conditioned behaviours (CRs) via LA's targets. The basolateral nucleus of the amygdala (BL) receives LA inputs and projects to various subcortical sites that can drive aversive and appetitive CRs. Consistent with this, BL neurons also develop increased responses to CSs that predict rewarding or aversive outcomes. This increased BL activity is thought to reflect the potentiated sensory responses of LA neurons. Here we contrast the CS-related activity of BL neurons when rats produced the expected CR or not, to show that cells activated by appetitive CSs mainly encode behavioural output, not CS identity. The strong dependence of BL activity on behaviour irrespective of CS identity suggests that feedforward connectivity from LA to BL can be overridden by other BL inputs.

## Abstract (web summary)

Neurons in the basolateral amygdala show increased response to conditioned stimuli predicting rewards. Here the authors show that their activity is not correlated with the reward-predicting stimulus but rather with the conditioned behaviour.

## Graphical abstract

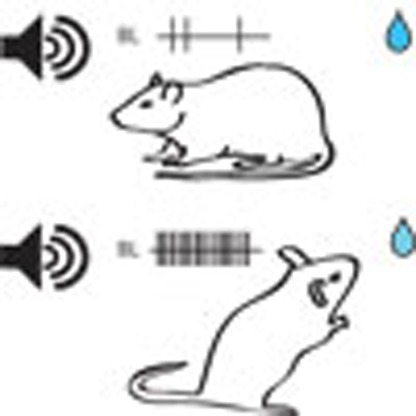

The amygdala is critical for the acquisition of conditioned responses (CRs) to stimuli (CSs) that predict aversive^1^ or rewarding outcomes^2^,^3^. As the amygdala's main recipient of sensory inputs from the thalamus and cortex^1^, the lateral nucleus (LA) is ideally positioned to store associations between CSs and unconditioned stimuli (USs). Consistent with this, many LA neurons acquire robust responses to positively or negatively valenced CSs as a result of conditioning^4^,^5^,^6^,^7^,^8^,^9^,^10^. Moreover, *ex vivo* studies have shown that conditioning alters the efficacy of thalamic and cortical synapses thought to convey CS information to LA neurons^11^,^12^,^13^,^14^,^15^. Together, these and other findings led to the view that the changes in CS responsiveness displayed by LA neurons following appetitive or aversive conditioning constitute potentiated sensory responses that drive approach or defensive CRs via different downstream neuronal populations targeted by LA.

One of the main recipients of LA inputs is the basolateral nucleus (BL) of the amygdala^16^,^17^. BL is a good candidate for mediating LA influences over different CRs. Indeed, different subsets of BL neurons could drive a variety of aversive and appetitive CRs via their divergent projections to the central medial amygdala^17^,^18^,^19^, bed nucleus of the stria terminalis^20^, nucleus accumbens^21^,^22^ or the dorsolateral striatum^22^,^23^. Consistent with this, BL neurons also show increased responses to CSs that predict rewarding or aversive outcomes as a result of conditioning^3^,^24^,^25^,^26^,^27^,^28^. Moreover, BL inactivation, lesion or optogenetic inhibition impair the acquisition or expression of aversive and appetitive CRs^3^,^29^,^30^,^31^,^32^,^33^,^34^.

However, a recent study revealed that BL activity during foraging is closely linked to behavioural output, not to reward or threat contingencies^35^. This finding raises the possibility that the increased CS-responsiveness of BL neurons in conditioning tasks is not directly driven by the potentiated sensory responses of LA neurons, but instead reflects training-induced modifications in behavioural output. According to this view, the enhanced CS-responsiveness observed previously in BL resulted from the increased probability that the CS would elicit a CR after conditioning. Under this hypothesis, BL neurons would appear to respond to the CS when in fact they only fire when the CR is emitted. The present study examined this possibility by comparing the activity of BL neurons when rats emitted the CR or not. This revealed that the CS-related firing of BL neurons varies strongly with conditioned responding. Therefore, the activity of BL neurons mainly encodes behavioural output.

## Results

### Overview of the conditioning paradigm

**Figure 1:**
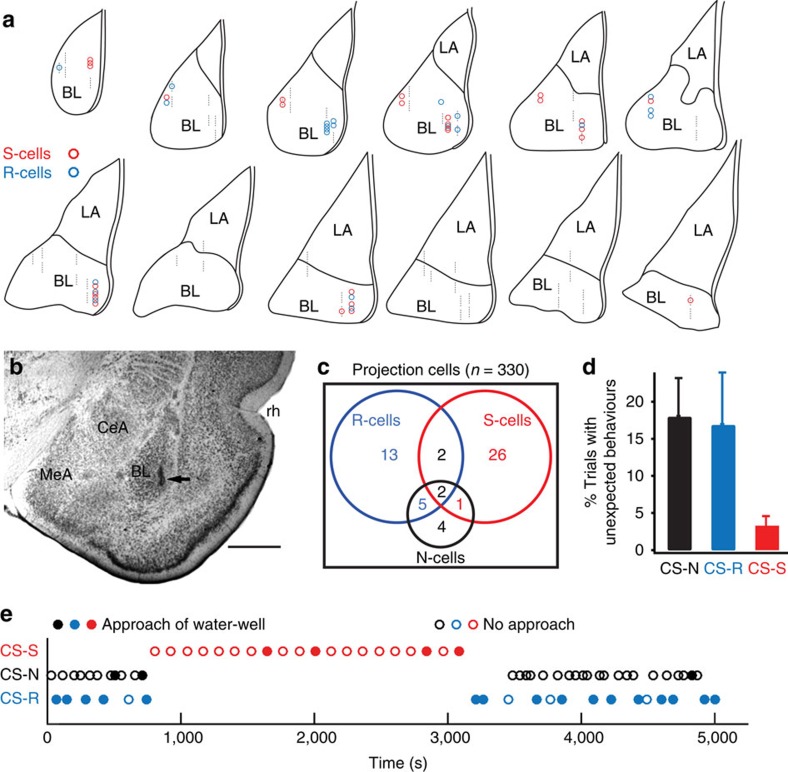
Experimental paradigm. (**a**) Schemes showing location of cells excited by the CSs (coloured circles). Dashed lines: recording sites where no cells excited by the CSs were detected. Cells excited only by the CS-N are not shown. Cells excited by the CS-R and other CS types are indicated as R-cells. (**b**) Coronal section with electrolytic lesion (arrow) marking recording site. Scale bar, 1 mm. (**c**) Venn diagram summarizing the CS responsiveness of projection cells. (**d**) Proportion of trials with unexpected behaviours emitted during the three CSs (average±s.e.m.; five rats). (**e**) Timing (*x* axis) of CS presentations (*y* axis) in one rat. Filled and empty circles: trials where CS triggered approach of water-port or not. CR-R, CS predicting reward; CS-N, neutral CS; CS-S, CS predicting footshock; N-Cells, R-Cells and S-Cells are cells with significantly increased firing rates during the CS-N, CS-R and CS-S, respectively.

To test whether the increases in CS-responsiveness induced by conditioning are related to changes in conditioned behaviour or driven by the CS, we recorded basolateral amygdala (BL) neurons (Fig. 1a,b) in rats trained on a mixed appetitive and aversive learning paradigm and contrasted their activity when rats produced the expected CR or not. Three different CSs were presented (white noise, 4 or 12 kHz tone; counterbalanced across subjects). During training, one CS (CS-R) predicted delivery of sweetened water, another (CS-S) was paired to a footshock, and the last (CS-N) was followed by a neutral, dim-light stimulus. Rats (*n*=5) were first trained to retrieve a liquid reward at the conclusion of the CS-R, not the CS-N. Then, they were implanted with multi-shank silicon probes in BL. After recovery from the surgery, they were fear conditioned. The data described below were obtained ⩾24 h later, when rats were presented with the three CSs in the same recording sessions. During these sessions, CS-Rs continued to predict reward delivery to prevent extinction. In contrast, CS-S presentations were unreinforced because pilot tests revealed that continuous reinforcement led to persistent freezing, interfering with CS-R responding.

### Activity of BL neurons during CSs of different valence

BL neurons (*n*=356) were classified as projection cells (*n*=330) or interneurons (*n*=26) based on firing rate and spike duration. This report focuses on projection cells. To assess whether CS-related changes in firing rates were significant, each cell's activity was binned (0.5 s) and a Wilcoxon rank-sum test was used to compare firing rates before versus during the CS (0.005 significance threshold). Overall, 16% of projection cells showed significant increases in firing rates during at least one of the CSs (CS-R, 6.7%; CS-S, 9.4%; CS-N, 3.6%; Fig. 1c). Very few cells were selectively responsive to the CS-N (1.2%). Only 1.2% of projection cells exhibited significant excitation during both the CS-R and CS-S, indicating that largely separate population of neurons respond to these two CSs. Below, cells with significantly increased activity during the CS-R or CS-S are termed R-cells and S-cells, respectively.

### CS-related activity of BL neurons varies with behaviour

To test whether the activity of responsive cells is determined by CS identity or the CRs they elicit, we compared trials where rats emitted the expected CR or not in response to the same CS. The following responses were defined as ‘unexpected': water-port approach to the CS-S or CS-N; no approach of the water-port in response to the CS-R. Behavioural responses on all other trials were considered as expected. Trials with unexpected behavioural responses were ∼5 times more frequent in response to the CS-R and CS-N than the CS-S (Fig. 1d). Figure 1e shows the session with the highest proportion of CS-S trials with unexpected approach behaviours.

Whereas trials with unexpected behaviours were seen in all rats with the CS-R or CS-N, they occurred in only two rats with the CS-S. Moreover, CS-S trials with unexpected approach behaviour occurred late in the session (trial 16±2.8), thus after the development of fear extinction. Since depotentiation of synapses conveying CS information was previously implicated in extinction^36^, such trials cannot be used to determine whether S-Cells encode behaviour or CS identity. Besides, the significance of motor responses (or lack of) elicited by the CS-S is ambiguous because they could represent active or passive defensive behaviours (for example, escape versus freezing). Consequently, this report will focus on R-cell activity in relation to the CS-R and CS-N.

**Figure 2:**
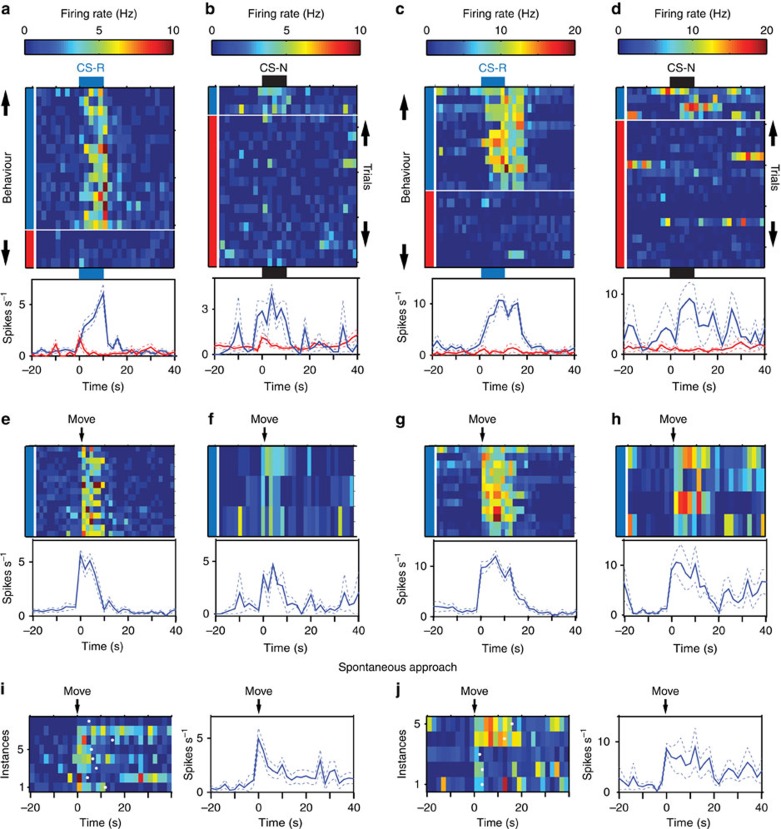
Representative examples of R-cells. The left**-** (**a**,**b**,**e**,**f**,**i**) and right-most (**c**,**d**,**g**,**h**,**j**) panels show two different cells. Responses elicited by CS-R (**a**,**c**) or CS-N (**b**,**d**). For each cell and CS combination, activity is depicted as a colour-coded raster (top) and peri-stimulus histogram of neuronal discharges (bottom; solid and dashed lines are averages and s.e.m., respectively). Trials are grouped by behaviour (blue, approach of water-port; red, no approach; bar on left of rasters). Rasters and peri-stimulus histograms in (**e**–**h**) depict the activity of the same cells as in (**a**–**d**) but aligned to onset of water-port approach instead of CS onset. Trials in (**e**–**h**) were obtained with the CS-R and CS-N, respectively. (**i**,**j**) Rasters and histograms show activity of the cells when rats spontaneously initiated approach of the water-port in the absence of CS. White dots in rasters shown in (**i**,**j**) indicate time when rats left the water-port. Approach initiation time was defined as the time point when rats initiate their final approach movement with straight trajectory and no pause towards water-port (see details in Methods section). Departure from water-port was defined as time when rats head moved away from the water-port for at least 2 s.

Figure 2a–d depicts the activity of two R-cells from different rats during the CS-R and CS-N, separating approach and no approach trials. On CS-R trials where rats approached the water-port, the CS-related activity of R-cells was strong (Fig. 2a,c; blue), but it was weak (Fig. 2a, red) or absent (Fig. 2c, red) when rats omitted the CR. Strikingly, when rats approached the water-port in response to the CS-N, R-cells fired strongly (Fig. 2b,d; blue), even though they normally showed little or no activity during this CS (Fig. 2b,d; red). Importantly, the firing rate increases seen during expected and unexpected approach trials were more closely tied to the initiation of approach behaviours (Fig. 2e–h) than to the onset of the CS-R or CS-N (Fig. 2a–d). BL neurons also increased their firing rates during spontaneous approach of the reward port (Fig. 2i,j).

**Figure 3:**
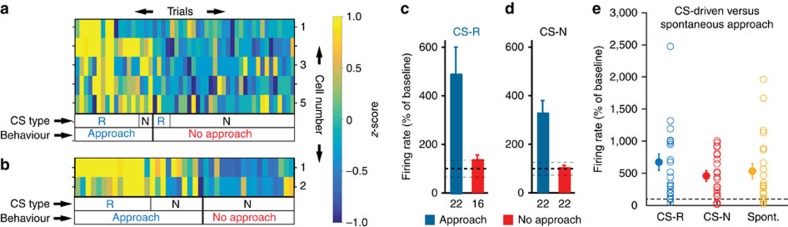
Dependence of R-cell activity on behaviour and CS identity. (**a**,**b**) Two simultaneously recorded sets of R-cells from different rats. Each horizontal line is a different cell. Each bin represents the average firing rate of a cell during a CS and trial (average – baseline; *z*-scored within each cell to visually harmonize the different cells). Trials (columns) are grouped by behaviour (legend at bottom) and CS identity (next to bottom row). (**c**,**d**) Average±s.e.m. firing rate of all R-cells during presentation of the CS-R (left) and CS-N (right). Numbers below histograms indicate sample size. Firing rates were normalized to baseline activity (solid and dashed lines: average±s.e.m. baseline firing rates). (**e**) Firing rate of R-Cells when rats approached the water-port during the CS-R (blue), the CS-N (red) or spontaneously (yellow). Firing rates were integrated over a 2 s window starting at the moment of approach initiation. Empty symbols, individual cells. Filled symbols, average±s.e.m. Firing rates were normalized to mean baseline (pre-CS) activity (horizontal dashed line). For approach behaviours in response to the CS-R, to avoid contamination of US (drinking)-related activity, periods after CS-offset were excluded. This reduction in the duration of the available period was compensated for in the firing rate calculation.

The same behavioural dependence is apparent in Fig. 3a,b, which shows sets of R-cells recorded simultaneously in two different rats and in Fig. 3c,d, which contrasts the average responses of all recorded R-cells during approach (blue) versus no-approach (red) trials with the two CS types. A two-way analysis of variance (ANOVA) on the responsiveness of R-cells revealed a significant effect of behaviour (*F*_(1,78)_=155.60, *P*<0.0001) but not CS identity (*F*_(1,78)_=2.03, *P*=0.16) and no interaction (*F*_(1,78)_=9.84, *P*=0.27). The proportion of variance explained by behaviour (17.8%) was much higher than that explained by CS identity (1.0%).

Last, we compared the magnitude of the firing rate increases displayed by R-cells in relation to water-port approach behaviours that occurred in response to the CS-R, CS-N or spontaneously (5.1±0.8 per rat; Fig. 3e). However, a repeated measure ANOVA detected no significant effect of condition (F_(2,63)_=1.55, *P*=0.22) suggesting that R-Cells increase their firing rates to a similar degree in relation to approach behaviours observed during the CS-R, CS-N or spontaneously. The increased firing rate of R-cells during spontaneous approach behaviours further supports the notion that they encode behavioural output.

## Discussion

The present study examined whether BL neurons always respond to appetitive CSs irrespective of whether CRs are emitted versus whether BL neurons only fire when the CR is emitted. The interest of this question stems from prior studies indicating that appetitive conditioning leads to a potentiation of LA responses to the CS-R, which in turn would automatically drive CRs via its targets, including the BL nucleus. Our results indicate that the CS-related firing of R-cells varies strongly with conditioned responding: it is absent or weak when rats omit the CR, present when they emit it and associated with the CS-N when rats mistakenly approach the water-port. Therefore, the activity of R-cells encodes behavioural output; their activity during the CS-R is not an obligatory consequence of the potentiated sensory responses of LA neurons to the CS-R. Given that many prior pharmaco-behavioural and optogenetic studies have established that BL activity plays a causal role in the genesis of conditioned appetitive behaviours^3^,^26^,^28^,^30^,^32^,^33^,^34^, our findings imply that BL activity contributes to drive conditioned behaviours; their CR-related activity is not a mere efferent copy. At present, it is unclear whether the responses of BL neurons to aversive CSs also show a strong relation to behaviour. Indeed, the present experimental design proved unsuitable to probe this question because of the confounding influence of extinction, and behavioural ambiguity. Settling this issue will require an altogether different behavioural paradigm where aversive CSs are continuously reinforced to prevent the development of extinction, and testing conditions where active and passive defensive behaviours can be distinguished from each other and from lack of CRs.

At present, it appears unlikely that the strong behavioural dependence of neuronal activity we observed in BL also applies to LA, the entry point of most sensory afferents into the amygdala^1^. Indeed, prior work^4^,^5^,^6^,^7^,^8^,^9^,^10^,^11^,^12^,^13^,^14^,^15^ suggests that in LA, CS-evoked responses are closely tied to the sensory properties of CSs and relatively independent of behaviour. These considerations raise the question of how a stimulus-value code in LA is transformed into a behavioural response code in BL. Foremost is the glutamatergic feedforward connectivity from LA to BL. According to this model, LA neurons that encode CSs with particular valences would form excitatory synaptic contacts with matched effector neurons in BL, resulting in the emission of the appropriate behaviour once the response of the relevant subset of LA neurons is potentiated. Consistent with this possibility, we observed that some cells showed an initial, behaviour-independent, increase in activity at CS-R onset (Fig. 2a; red trace).

By itself, however, this feedforward model appears insufficient to explain the strong correspondence between BL activity and behaviour irrespective of stimulus identity. To account for the observation that BL neurons do not fire during the CS-R when animals omit the CR (and reciprocally for the CS-N), other pathways such as those arising from prefrontal and medial temporal cortices^37^ or midline thalamic nuclei^38^ should exist to override the signal emanating from LA. Within BL, spiking is regulated by an extensive local recurrent inhibitory network^39^,^40^,^41^,^42^. In particular, the two main subtypes of inhibitory interneurons in BL, the parvalbumin and somatostatin expressing interneurons, are differentially innervated by cortical inputs. Parvalbumin cells receive little if any cortical inputs^39^, whereas somatostatin cells are strongly innervated by cortical inputs^40^. Thus, in addition to their direct excitatory influence over projection cells, cortical inputs exert, via the activation of somatostatin interneurons, a disynaptic inhibitory influence, which could reduce or suppress the responses of projection cells to other excitatory inputs, including LA inputs about the CS. In contrast, suppression or disfacilitation of somatostatin cells would allow BL projection cells to fire in response to other inputs and trigger reward approach behaviours. Conceivably, this intrinsic inhibitory network might differentially regulate activity in distinct ensembles of behaviour-coding BL neurons. Of course, the above mechanism awaits experimental scrutiny.

Another open question relates to the significance of the increased firing rate of R-cells in relation to approach initiation. As suggested previously, the activity of these cells might encode components of learned reward-seeking behaviours like reward expectation^43^,^44^. Such signals might be important not only for driving reward-seeking behaviour^3^,^30^,^32^,^33^,^34^ but also for memory maintenance and updating^43^,^45^,^46^.

## Methods

Procedures were approved by the Institutional Animal Care and Use Committee of Rutgers University, in compliance with the Guide for the Care and Use of Laboratory Animals (DHHS). We used male naïve Sprague-Dawley rats (310–360 g at the beginning of experiments, Charles River Laboratories, New Field, NJ) maintained on a 12 h light/dark cycle. All experiments were performed during the light cycle.

### Overview of the experimental timeline

After habituation to the animal facility and handling, five rats were subjected to a food restriction protocol. Then, they received daily training sessions in the reward-seeking task. After they mastered the task, they were implanted with multi-shank silicon probes in the BL nucleus. Following recovery from the surgery, they were re-trained on the reward-seeking task. Once they regained their proficiency, rats were fear conditioned. BL neurons were recorded while rats were presented with the various CSs in the same session.

### Behavioural apparatus

The conditioning chamber for the appetitive task (context A) was a rectangular box made of black polyvinyl chloride (width, length and height: 28, 29 and 38 cm, respectively) located inside a sound-attenuating box (Coulbourn Instruments). One wall featured a water-port located 8–10 cm above the floor. Its height was adjusted for each rat to minimize random water-port visits. The chamber was dimly illuminated (∼2 Lux) by a house light located 29 cm above the floor and a small light near the water-port. An additional house light protected by transparent Plexiglas was installed over the water-port zone to be used as neutral unconditioned stimulus. Two digital videocameras recorded the rats' behaviours: one located above the water-port and the other above the center of the conditioning chamber. Two speakers were attached to the ceiling.

Fear conditioning was performed in a rodent conditioning chamber (context B) with a metal grid floor and aluminium and Plexiglas wall that was enclosed within a sound-attenuating box. The chamber was dimly illuminated by a single house light.

### Reward conditioning

Rats were trained to associate two CS (CS-R or CS-N) with a rewarding US (20% (w/v) sucrose solution; 30 μl) or neutral US (2 s continuous light), respectively. To ensure proper motivation during conditioning, daily access to food was restricted so that the rats' body weight was maintained at ∼85% of their free-feeding weight. In each rat, the CS-R and CS-N were randomly selected (using a random number generator) among three different pulsing sounds at 75 dB, each lasting for 10 s: 4 kHz (200 ms on, 200 ms off), 12 kHz (1,400 ms on, 200 ms off) and white noise (1,000 ms on, 400 ms off). The third sound was used for fear conditioning as described below. The investigator was aware of the identity of the various CSs.

Termination of the CS-R or CS-N coincided with delivery of the reward or light US, respectively. The sucrose solution was dispensed in the water-port using a solenoid pinch valve. If not ingested by the rat, it was removed from the port by a peristaltic pump 7 s after delivery. Initially, rats were trained with the CS-R only (60 min daily training sessions with 30 trials each; pseudorandomly with average intertrial interval of 120 s). Rats that reached criterion (consumed ⩾80% of rewards) were further trained for discrimination between the CS-R and CS-N until water-port approach ratio in response to the CS-R became four times higher than that seen in response to the CS-N. Rats that successfully discriminated the two CSs (*n*=5) next underwent stereotaxic surgery.

### Surgery

Rats were anaesthetized with a mixture of isoflurane and O_2_, and administered atropine sulfate (0.05 mg kg^−1^, i.m.) to aid breathing. In aseptic conditions, rats were mounted in a stereotaxic apparatus with non-puncture ear bars. A local anaesthetic (bupivacaine, sc) was injected in the scalp. Fifteen minutes later, the scalp was incised and a craniotomy performed above the amygdala. Then, 64-channel multi-shank silicon probes (Buzsaki64L, Neuronexus, Ann Arbor, MI) were stereotaxically aimed at the BL using the following coordinates: anteroposterior −2.2 to −3.6, mediolateral 5 to 5.3 and dorsoventral 8.4 (in mm, relative to bregma). Silicon probes consisted of eight shanks (inter-shank distance of 200 μm), each with eight recording leads (de-insulated area of 160 μm^2^) separated by ∼20 μm dorsoventrally. They were attached to microdrives^47^, allowing us to modify their position. Rats were allowed 2–3 weeks to recover from the surgery.

### Fear conditioning

After recovery from the surgery, rats were re-trained to criterion in the appetitive task over 2–3 days. Then, on two consecutive days, rats were first habituated to the CS-S (five unpaired presentations in context A) and then to context B (20 min; no CS-S). On the next day, while in context B, rats received four additional unpaired CS-S presentations followed by five presentations of CS-S (20 s), each immediately followed by a foot shock (0.5 mA, 1 s). On the next day, we verified that conditioned fear responses had been acquired. Because pilot tests revealed that fear conditioning interfered with responsiveness to the CS-R, on the next 1–4 days, rats were again trained on the appetitive portion of the task, as needed for their performance to reached criterion.

### Analysis of approach and departure from water-port

The rats' behaviour was recorded by an overhead videocamera and a second camera installed in front of water-port (frame rate, 10 Hz). Our analyses are based on a frame-by-frame analysis of behaviour and video analysis was done manually. Scoring of behaviour was performed blind to the corresponding unit activity. Approach behaviour was scored as positive when the rat positioned its head over the water-port during a 1 s time window before CS termination or remained there for >2 s during the CS period. When referencing peri-event time histograms of neuronal discharges to behaviour rather than CS onset, approach behaviours were divided into two phases: initial slow approach and final approach. Indeed, when the CS-R was presented, rats often initially made a relatively slow approach towards the water-port from their initial sitting position. This slow approach usually involved several body turns with one or more pauses. As a result, the onset of this slow approach often could not be identified unambiguously. However, rats eventually made a quick final approach movement towards the water-port. Typically, this final approach could be unambiguously identified because it was characterized by a straight and uninterrupted trajectory. Thus, we used the onset time of this final approach in our neuronal analyses. Usually, the rats' snout reached water-port within a second after onset of this final approach.

For analyses of neuronal activity around spontaneous approach behaviours, we considered only water-port approaches that were initiated in the absence of CSs and met the following criteria. First, rats stayed at the water-port more than 2 s after port-entry. Second, the spontaneous approach was not preceded by another spontaneous approach for at least for 10 s. Otherwise, the onset of spontaneous approach was identified as described above for the case of CS-induced approach (approach towards water-port with straight trajectory and without pause).

### Recording and analysis

On the next day, while recording behaviour and BL unit activity, rats were presented with the three CSs in context A, as shown in Fig. 1e. Except where indicated, presentations of the CS-R were reinforced to prevent extinction. In contrast, the CS-S was not because pilot tests revealed that paired presentations led to persistent freezing, interfering with CS-R responding. Recording sessions featured four stages. First, rats were presented with a random mixture of 6–12 CS-Rs and CS-Ns, using a variable inter-trial interval (mean of 110 s for the CS-R). Second, rats received 20–23 presentations of the CS-Ss (inter-trial interval of 100 s). Third, rats were again presented with a mixture of CS-Rs and CS-Ns. Fourth, rats again received CS-R and CS-N presentations, but the rewarding US was omitted after the CS-Rs. CS-R extinction trials were not considered in this study. In phases 1, 3 and 4, CS-Ns were ∼1.6 times more frequent than CS-Rs. Most of the data included in this study were obtained in phases 1–3 of the above recording session (68% of R-cells and 71% of S-cells). However, to increase sample sizes, a second recording session was performed in a subset of rats. Before this session, rats were re-trained on the fear conditioning and reward tasks on subsequent days. Silicon probes were lowered or raised ⩾140 μm before the recordings. A similarly strong dependence of R-cell activity on behaviour was observed in both recording sessions.

Signals were sampled at 25 kHz and stored on a hard drive. The data was first high-pass filtered using a median filter (window size of 1.1 ms), then thresholded to extract spikes. We next ran PCA on the spikes. Using KlustaKwik (http://klustakwik.sourceforge.net/), spikes were automatically sorted into single units using the first three components from each electrode (eight by shank for a total of 24 components). Spike clusters were then refined manually using Klusters^48^. The reliability of cluster separation was verified by inspecting auto- and cross-correlograms. Autocorrelograms had to display a refractory period of at least 2 ms. Crosscorrelograms should not show evidence of a refractory period, as this feature betrays overlap between clusters. Units with unstable spike shapes during a given recording session were excluded.

To determine spike duration, we first selected the channel where, for a given cell, action potentials had the largest peak to trough amplitude. We then measured the spikes duration as the time between spike trough and peak^49^. BL cells were classified as presumed projection cells or interneurons on the basis of their baseline firing rates (cutoff 2.5 Hz) and spike duration (spike through to peak of 0.5 ms).

### Histology

At the end of the experiments, rats were anaesthetized with isoflurane. On each shank, one of the recording sites was marked with a small electrolytic lesion (10 μA between a channel and the animals' tail for 10 s). One day later, under deep isoflurane anaesthesia, rats were perfused-fixed through the heart, their brains extracted, cut on a vibrating microtome and the sections counterstained with cresyl violet. We only considered neurons that were histologically determined to have been recorded in BL.

### Statistical analyses

All data are reported as average±s.e.m. All statistical tests were two-sided. In all cases, all available cells, trials and subjects were included in the statistical analyses, as appropriate. To access the significance of firing rate changes of individual cells during CS presentation, we binned (0.5 s) the baseline period (20 s before CS onset) and CS period, then calculated average bin values across trials for each bin. All available trials were used for the CS-R and CS-N. For the CS-S, only the first six trials were used to avoid extinction. Then, we compared averaged bin values of the baseline period and CS period using a rank-sum test with a significance threshold of *P*<0.005. Distributional assumptions were met for this non-parametric test. It should be noted that the number of cells whose CS-related activity was deemed significant with a threshold of 0.005 did not change appreciably with more liberal significance thresholds. For instance, only two additional R-Cells were detected with a *P* of 0.05 and they were not responsive to the CS-S.

For the group statistical analysis of Fig. 3, because we used an unbalanced and multifactorial design, we had to use two-factor (behaviour and CS identity) ANOVA with an interaction term. However, we verified the conclusions of this analysis using a non-parametric ANOVA for balanced designs (Friedman test). To adapt our data to this test's requirements, we excluded cells devoid of CS-R no approach trials so that the n's in all groups would be the same. As for the parametric ANOVA, the Friedman test revealed a strong effect of behaviour (*χ*^2^=20.29, *P*<0.0001) and no effect of CS identity (*χ*^2^=1.22, *P*=0.2687). The proportion of variance explained by behaviour and CS identity was calculated using the omega squared method: (SSfactor−(df *MSE))/(SST+MSE) where SSFactor stands for sum of squares for factor, MSE for mean square error, SST for sum of squares total and df for degree of freedom of factor.

### Data availability

The data that support the findings of this study are available from the corresponding author on request.

## Additional information

**How to cite this article:** Lee, S.-C. *et al*. Basolateral amygdala nucleus responses to appetitive conditioned stimuli correlate with variations in conditioned behaviour. *Nat. Commun.* 7:12275 doi: 10.1038/ncomms12275 (2016).
